# Validation of the maximal cardiopulmonary exercise test in adolescents with major depressive disorder and comparison of cardiorespiratory fitness with sex- and age-related control values

**DOI:** 10.1007/s00431-023-05304-6

**Published:** 2023-10-31

**Authors:** Charlotte Wenzel, Bart Chateau Bongers, Marit Lea Schlagheck, Daniela Reis, Franziska Reinhard, Peter Schmidt, Stefan Bernitzki, Max Oberste, Heidrun Lioba Wunram, Philipp Zimmer, Oliver Fricke

**Affiliations:** 1 Division of Performance and Health, Institute for Sport and Sport Science, TU Dortmund University, Dortmund, Germany; 2 Department of Nutrition and Movement Sciences, Faculty of Health, Medicine and Life Sciences, Maastricht University, School of Nutrition and Translational Research in Metabolism (NUTRIM), Maastricht, the Netherlands; 3 Department of Surgery, Faculty of Health, Medicine and Life Sciences, School of Nutrition and Translational Research in Metabolism (NUTRIM), Maastricht University, Maastricht, the Netherlands; 4 Institute for Integrative Medicine, Witten/Herdecke University, Professorship for Integrative Pediatrics, Witten, Germany; 5 Department of Pediatrics, Gemeinschaftskrankenhaus Herdecke gGmbH, Herdecke, Germany; 6 Department of Child and Adolescent Psychiatry, Psychosomatics and Psychotherapy, University Hospital of Cologne, Cologne, Germany; 7 Department of Special Care Dentistry, Witten/Herdecke University, Faculty of Health, Witten, Germany; 8 Department of Pediatrics, Evangelisches Krankenhaus Düsseldorf, Düsseldorf, Germany; 9 Institute of Medical Statistics and Computational Biology, University of Cologne, Medical Faculty and University Hospital of Cologne, Cologne, Germany; 10 Department of Pediatrics, University Hospital of Cologne, Cologne, Germany; 11 Department of Human Medicine , Witten/Herdecke University, Faculty of Health, Witten, Germany; 12 Department of Child Adolescent Psychiatry and Psychotherapy, Klinikum Stuttgart, Stuttgart, Germany

**Keywords:** Exhaustion criteria, Maximal oxygen consumption, Aerobic fitness, Cycle ergometer, Control values

## Abstract

Endurance training has been shown to be effective in treating adolescents with major depressive disorder (MDD). To integrate endurance training into the therapeutic setting and the adolescents' daily lives, the current performance status of the adolescents should be accurately assessed. This study aims to examine adolescents with MDD concerning exhaustion criteria during a cardiopulmonary exercise test (CPET), as well as to compare the values obtained thereon with sex- and age-related control values. The study included a retrospective examination of exhaustion criteria ((i) oxygen consumption (V̇O_2_) plateau, (ii) peak respiratory exchange ratio (RER_peak_) > 1.0, (iii) peak heart rate (HR_peak_) ≥ 95% of the age-predicted maximal HR, and (iv) peak blood lactate concentration (BLC_peak_) > 8.0 mmol⋅L^−1^) during a graded CPET on a cycle ergometer in adolescents with MDD (n = 57). Subsequently, maximal V̇O_2_, peak minute ventilation, V̇O_2_ at the first ventilatory threshold, and peak work rate of participants who met at least two of four criteria were compared with published control values using an independent-sample t-test. Thirty-three percent of the total population achieved a V̇O_2_ plateau and 75% a RER_peak_ > 1.0. The HR and BLC criteria were met by 19% and 22%, respectively. T-test results revealed significant differences between adolescents with MDD and control values for all outcomes. Adolescents with MDD achieved between 56% and 83% of control values.

*   Conclusions*: The study shows that compared with control values, fewer adolescents with MDD achieve the exhaustion criteria on a CPET and adolescents with MDD have significantly lower cardiorespiratory fitness.

*   Clinical trial registration*: No. U1111-1145–1854.

**Table:** 

**What is Known:** *• It is already known that endurance training has a positive effect on depressive symptoms.*	
**What is New:** *• A relevant proportion of adolescents with major depressive disorder do not achieve their V̇O2max during a graded cardiopulmonary exercise test.* *• Adolescents with major depressive disorder have significantly lower cardiorespiratory fitness compared to sex- and age-related control values.*

**What is Known:**

*• It is already known that endurance training has a positive effect on depressive symptoms.*

**What is New:**

*• A relevant proportion of adolescents with major depressive disorder do not achieve their V̇O2max during a graded cardiopulmonary exercise test.*

*• Adolescents with major depressive disorder have significantly lower cardiorespiratory fitness compared to sex- and age-related control values.*

## Introduction

Physical inactivity increases the risk for various diseases including major depressive disorder (MDD) [1]. Especially during adolescence, social, physical, and psychological changes significantly raise the incidence of MDD [2]. During this period of life, only around 20% of individuals are sufficiently physically active and meet the World Health Organization (WHO) recommendation of an average of 60 min of moderate to vigorous physical activity daily [3]. To fulfill this recommendation and counteract symptoms of MDD, a strong social network, and a supportive family relationship can positively influence participation in extracurricular activities [4, 5].

Beyond that, evidence from meta-analyses further suggests that targeted exercise therapy counteracts MDD in both, adults and adolescents [6–9]. Since physical activity is difficult to measure objectively, cardiorespiratory fitness (CRF) is often assessed as a quantifiable factor influencing MDD [10]. Low CRF is related with a 75% increased risk of developing depression [1]. CRF is associated with the ability to perform dynamic exercise at high muscle strength and moderate to high intensity for prolonged periods of time [11]. It is also connected to a healthy body composition, which in turn may reflect improved body satisfaction, higher self-esteem, and better social behavior. These factors can lead to lower depressive symptoms [5, 12, 13].

Clinical trials for the treatment of MDD have focused on endurance training on a treadmill or cycle ergometer, as aerobic exercise improves depressive symptoms the most [9, 14]. The cardiopulmonary exercise test (CPET) on a cycle ergometer is considered the gold standard to provide valid statements about individual CRF by direct measurement of maximal oxygen consumption (V̇O_2max_). In addition to respiratory gas analysis, CPET usually includes the recording of heart rate (HR) and measuring blood lactate concentration (BLC), as well as a rating of perceived exertion [11]. CPET is a useful approach to assess current CRF, determine appropriate training intensity for therapeutic purposes, and increase motivation to incorporate physical exercise training into daily life. The American College of Sports Medicine (ACSM) suggests exhaustion criteria for a CPET in adults to designate results obtained as maximal and valid. These criteria still need to be adapted for children and adolescents, as there is no definite consensus yet [11]. Against this backdrop, the following modified criteria were used in this study based on published literature: (i) reaching a V̇O_2_ plateau [15, 16], (ii) a peak respiratory exchange ratio (RER_peak_) > 1.0 [17], (iii) attainment of a peak HR (HR_peak_) ≥ 95% of the age-predicted maximal HR [18], and (iv) a peak BLC (BLC_peak_) > 8.0 mmol⋅L^−1^ [11]. However, there is no consistency on the number of criteria to be met to confirm the validity of V̇O_2max_ results [19]. The aim of this secondary research is, first, to examine a population of adolescents with MDD concerning the exhaustion criteria for verifying V̇O_2max_ on a CPET and, second, to compare the collected values with published sex- and age-related control values.

## Materials and methods

### Participants

The study included medication-naïve adolescents with diagnosed MDD who were treated as inpatients or day-clinic patients at the Department of Child and Adolescent Psychiatry, Psychosomatics, and Psychotherapy at the University Hospital of Cologne. The data were collected from 2013 to 2015 as part of an intervention study [14]. Participants had to meet international established criteria for nonpsychotic MDD (i.e. Diagnostic and Statistical Manual of Mental Disorders-Fourth-/-Fifth Edition (DSM-IV/-5) and International Classification of Diseases-10th Revision (ICD-10)) [20] and had a baseline score in *Depressionsinventar für Kinder und Jugendliche* (DIKJ) [21] of ≥ 18 raw points. In addition, they had to be of normal intelligence (Intelligence Quotient > 70 (Kaufman Assessment Battery for Children or Wechsler Intelligence Scale for Children)) and be proficient in German language and writing. Both biological sexes were considered.

Adolescents were excluded if they had any of the following conditions: schizophrenia, other psychotic disorders or psychoses, psychotic depression, bipolar disorders I and II, personality disorders, pervasive developmental disorders, or current substance abuse. A body mass index < 16 kg⋅m^−2^, diseases that result in a limitation of physical activity (e.g., insufficient gain of mass in eating disorders), malignant diseases, and morbus addison or unsubstituted hypothyroidism were also reasons for exclusion. In addition, the recorded values of CPET should appear to be valid. Written informed consent of the adolescents and their parents was required. The intervention study [14] on which this work is based was reviewed and approved by the ethics committee of the University Hospital of Cologne.

### CPET and data analysis

Participants performed a graded CPET in an upright position on a cycle ergometer (Schiller, ERG 910 plus/ ERG 911) at the University Hospital Cologne. In brief, the test started with a two-minute resting measurement before increasing the workload for 25 watts every two minutes until participants were not able to maintain the pedaling frequency above 60 rpm [22]. This was followed by a recovery phase of at least 2 min without watt load. Time to exhaustion (TTE) may vary and should ideally range between 8 and 12 min [23]. TTE was defined by the time of the CPET minus the rest phase and the recovery phase. The peak work rate (WR_peak_) was calculated according to the following formula when the last stage was not fully completed [24]:

$${WR}_{peak}={WR}_f+\frac t{120}\cdot25,$$

where WR_f_ is the value of the last complete workload (W), t is the time the last workload was sustained (s), and 25 is the power difference between the last two workloads (W). The respiratory gas exchange was measured continuously breath by breath by spiroergometry (ZAN®). Heart rate was monitored constantly through an ear clip. The following parameters were extracted from the CPET: V̇O_2_, minute ventilation (V̇E)_,_ HR, work rate (WR), respiratory exchange ratio (RER), V̇O_2_ at the first ventilatory threshold (VT1), and TTE. The highest recorded 30-s average that was attained during the CPET was considered as peak V̇O_2_ (V̇O_2peak_), peak V̇E (V̇E_peak_), HR_peak_, and RER_peak_. The VT1 value was ascertained using the V̇-slope method, which determines the slope of the linear relationship between V̇O_2_ and carbon dioxide production (V̇CO_2_) [25]. Relative values were determined as absolute values divided by body mass in kilogram. For the measurement of BLC and BLC_peak_, blood was collected at rest, after each stage and after reaching the WR_peak_.

### Exhaustion criteria for the CPET

According to the ACSM, the guidelines for a CPET for children and adolescents are the same as for adults. However, the physiological values differ [11]. Consequently, the criteria were selected based on these differences using the literature defined for children and adolescents. Four criteria for exhaustion during a CPET were examined. The first criterion was the achievement of a V̇O_2_ plateau. In this work, a plateau was defined as ≤ 150 mL⋅min^−1^ increase in V̇O_2_, during the last 30-s average of the penultimate stage to the last 30-s average of the final stage [15, 16]. The second criterion included a RER_peak_ > 1.0 based on published literature [17]. The third criterion was a HR_peak_ ≥ 95% of the age-predicted maximal HR. The equation of 208–0.7⋅age was used to calculate the predicted HR_peak_ [18, 26]. The fourth and last criterion was a BLC_peak_ > 8.0 mmol⋅L^−1^ [11]. ACSM additionally recommended a rating of perceived exertion at WR_peak_, which was not collected in this study.

### Statistical analysis

This is a secondary analysis of data from an intervention study [14]. All statistical analyses were evaluated with the Python programming language Python 3 [27] using Pandas [28], Matplotlib [29], Seaborn [30], and SciPy [31] packages. First, participant characteristics were presented by descriptive statistics (mean ± standard deviation (SD)). Second, achievement of the four criteria for maximal exhaustion was presented by sex using frequency and percentage. Third, descriptive statistics (mean ± SD) of CPET data from adolescents with MDD who met at least two of the four criteria were compared with published sex- and age-related control values [17]. Independent-sample Welch’s t-tests were performed to examine differences between the two groups. For all statistics, the level of statistical significance was set at p < 0.05. Cohen's d was used to estimate the effect size.

## Results

### Participants' characteristics

In total, n = 89 potential participants conducted CPET within the defined period. Finally, n = 57 (40 females; 17 males) nonmedicated adolescents with MDD aged 13 to 17 years met the requirements and were included in this analysis. Of the n = 31 excluded patients, n = 11 had a DIKJ < 18, n = 8 did not give consent, n = 6 were excluded for medical reasons, and n = 7 were excluded due to measurement errors during CPET or missing values. The anthropometric characteristics of the participants are shown in Table 1.

**Table 1:** Participants’ characteristics (n = 57)

	Female (n = 40; 70%)	Male (n = 17; 30%)	All (n = 57; 100%)
Age (years)	15.93 ± 1.18	16.06 ± 1.17	15.97 ± 1.16
Height (cm)	165.50 ± 5.80	177.53 ± 7.95	169.09 ± 8.50
Body mass (kg)	68.03 ± 16.84	72.39 ± 19.01	69.33 ± 17.46
BMI (kg⋅m^−2^)	24.85 ± 6.18	22.82 ± 5.27	24.24 ± 5.95
DIKJ score	29.19 ± 7.00	24.65 ± 3.67	27.74 ± 6.46

Data are presented as mean ± SD; BMI: body mass index, DIKJ: Depressionsinventar für Kinder und Jugendliche

### Proportion of the participants attaining the exhaustion criteria

The achievement of the exhaustion criteria is shown for the entire sample and both sexes in Table 2. The sample size varies depending on the criterion, as not all data could be collected completely. To achieve V̇O_2max_, a TEE between 8 and 12 min is optimal [23]. Since n = 2 participants were significantly below the recommended time, they were excluded from the plateau criterion. In addition, no lactate could be measured in n = 7 participants.

**Table 2:** Attainment of the criteria by the participants

	V̇O_2_ plateau (n = 55)		RER_peak_ > 1.0 (n = 57)		HR_peak_ ≥ 95% of the age predicted HR (n = 57)		BLC_peak_ > 8.0 mmol⋅L^−1^ (n = 50)		At least 2 out of 4 of the criteria (n = 57)	
	Yes (%)	No (%)	Yes (%)	No (%)	Yes (%)	No (%)	Yes (%)	No (%)	Yes (%)	No (%)
Overall sample	18 (33)	37 (67)	43 (75)	14 (25)	11 (19)	46 (81)	11 (22)	39 (78)	22 (39)	35 (61)
Sex										
Female	10 (26)	28 (74)	31 (77.5)	9 (22.5)	8 (20)	32 (80)	8 (23)	27 (77)	16 (40)	24 (60)
Male	8 (47)	9 (53)	12 (71)	5 (29)	3 (18)	14 (82)	3 (20)	12 (80)	6 (35)	11 (65)

Data are presented as counts (percentages); V̇O_2_: oxygen consumption; RER_peak_: peak respiratory exchange ratio; HR_peak_: peak heart rate, BLC_peak_: peak blood lactate concentration

### Comparison of CRF of adolescents with MDD with sex- and age-related control values

Subsequently, the CPET results of adolescents with MDD n = 22 (16 females; 6 males) who met at least two of the four criteria are compared with sex- and age-related control values [17]. The control values used as comparison for each adolescent with MDD were obtained from a cross-sectional observational study of n = 214 (100 females; 114 males) healthy participants aged 8 to 18 years. They performed a CPET on a cycle ergometer. After three minutes of rest, participants completed a three-minute unloaded warm-up period. Subsequently, the work rate was increased constantly by 10, 15, or 20 watts per minute, depending on participant’s body height. The children and adolescents cycled until they could no longer maintain a pedaling frequency of 60 rpm. This was followed by a five-minute unloaded recovery phase. A maximal effort was confirmed if one of the following criteria was met: a HR_peak_ > 180 beats⋅min^−1^ and/or a RER_peak_ > 1.0 [17]. Least-mean-squares methods [32] were used to determine sex- and age-related control values for each adolescent with MDD. The results are listed in Table 3. T-test results revealed significant differences between both groups for all CPET outcomes.

**Table 3:** Comparison of CRF

	Adolescents with MDD (n = 22)	Sex- and age-related control values	p-value	ES (d)
V̇O_2max_ (L⋅min^−1^)	1.84 ± 0.52	2.64 ± 0.31	< 0.001	1.87
V̇O_2max_ (mL⋅kg^−1^⋅min^−1^)	27.12 ± 6.94	43.74 ± 3.15	< 0.001	3.08
V̇E_peak_ (L⋅min^−1^)	68.91 ± 18.50	91.44 ± 10.28	< 0.001	1.51
V̇E_peak_ (L⋅kg^−1^⋅min^−1^)	1.03 ± 0.28	1.5 ± 0.11	< 0.001	2.21
V̇O_2_ at VT1 (L⋅min^−1^)	1.25 ± 0.4	1.51 ± 0.18	< 0.05	0.84
V̇O_2_ at VT1 (mL⋅kg^−1^⋅min^−1^)	18.25 ± 5.36	24.69 ± 2.03	< 0.001	1.59
WR_peak_ (W)	140.34 ± 37.98	218.82 ± 26.51	< 0.001	2.40
WR_peak_ (W⋅kg^−1^)	2.07 ± 0.47	3.68 ± 0.21	< 0.001	4.42

Data are presented as mean ± SD; ES = effect size determined using Cohen’s d; MDD: major depressive disorder; V̇O_2_: oxygen consumption; V̇O_2max:_ maximal oxygen consumption; V̇E_peak_: peak minute ventilation, WR_peak_: peak work rate; VT1: first ventilatory threshold

**Fig. 1:**
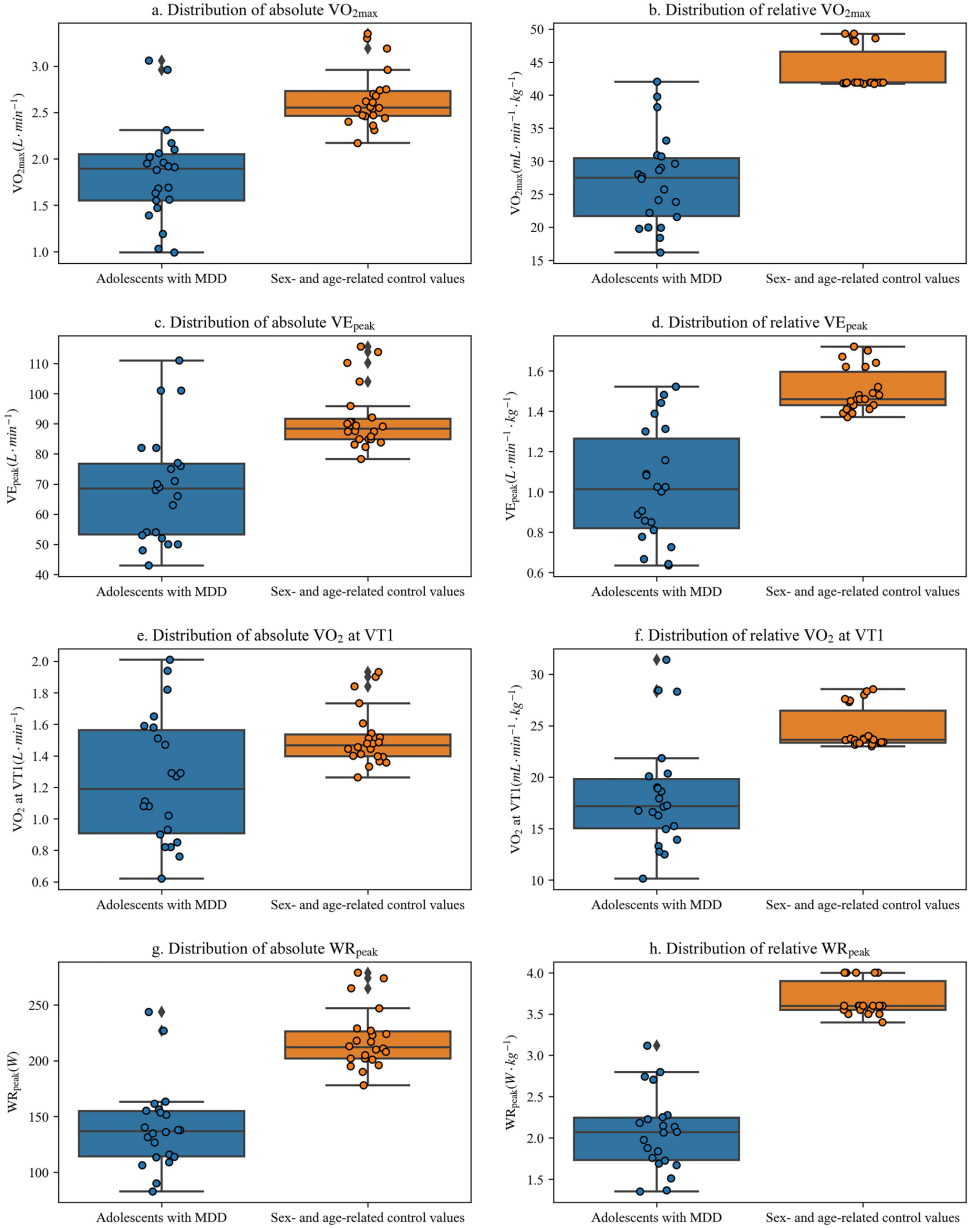
Distribution of CPET parameters of adolescents with MDD and sex- and age-related control values; MDD: major depressive disorder; V̇O_2_: oxygen consumption; V̇O_2_max: maximal oxygen consumption; V̇E_peak_: peak minute ventilation; VT1: first ventilatory threshold; WR_peak_: peak work rate

Figure 1(a-h) illustrates the distribution of CPET parameters. Adolescents with MDD achieved on average 70% of the absolute and 62% of the relative V̇O_2max_ of sex- and age-related control values. They reached 75% and 69% of the absolute and relative V̇E_peak_ of the control values, respectively. In addition, they attained 83% of the absolute and 74% of the relative V̇O_2_ at VT1 of the control values. Furthermore, adolescents with MDD achieved 64% of the absolute and 56% of the relative WR_peak_ of sex- and age-related control values.

## Discussion

The aim of this study was first to investigate the achievement of exhaustion criteria during a CPET in adolescents with MDD and second to compare the collected parameters with sex- and age-related control values. In this analysis, 39% of adolescents with MDD met at least two of the four exhaustion criteria, indicating the attainment of V̇O_2max_ and valid results for this population. Additionally, these participants attained 56%-83% of sex- and age-related control values for CRF, depending on the variable considered.

Reaching a V̇O_2_ plateau with increasing work rate is not commonly observed in CPET [11], particularly in children and adolescents [16]. This is also reflected in this analysis, in which a small proportion (33%) reached a V̇O_2_ plateau. This can be justified by the fact that patients with MDD have reduced CRF overall [33]. Another possible factor is that clinical populations as well as children and adolescents are often not motivated to perform maximal physical exertion [17].

The RER is considered a very accurate and objective parameter of individual exhaustion during a CPET [11], but is used inconsistently in the literature [34]. A RER_peak_ > 1.0 was met by a majority (75%), indicating that a large percentage of adolescents with MDD probably reached maximal cardiopulmonary exhaustion even if they did not reach a V̇O_2_ plateau. Moreover, a plateau is often lacking, especially in clinical populations, most likely due to the unpleasant symptoms of fatigue, dyspnea, leg discomfort, or a combination of these exercise limiting factors [17].

In contrast to the respiratory gas exchange outcome, the HR criterion was reached by only a small proportion (19%) of participants. Since the HR depends on various individual factors [35], this criterion is inconsistently defined in various studies [34]. Furthermore, previous studies showed that patients with MDD exhibit lower HR function and HR variability at rest and during stress [36, 37] due to hypo-reactivity [36]. Hypo-reactivity provides insight into the lack of cardiovascular change in patients with MDD that usually occurs in response to stress [33, 36].

Lactate is a metabolic product produced during vigorous physical activity [38]. Physical inactivity may be consequences of anhedonia, which typically occurs in patients with MDD [39]. This provides an explanation for the low percentage (22%) regarding the BLC criterion. In mice studies, lactate has even been shown to act as an antidepressant and enhance stress resistance [38]. Chronic stress may lead to an increased risk of developing MDD [40]. Another study comparing runners and non-runners showed a negative correlation between BLC and depression symptoms severity [41]. Accordingly, it might be that in the present study, adolescents with MDD have lower overall BLC due to their reduced CRF.

Compared to healthy adolescents, the proportion who attained maximal cardiopulmonary effort is significantly lower. Previous work report that 90% of healthy adolescents met the criteria [42]. The adolescents with MDD from this study who appropriately met the criteria were compared with sex- and age-related control values [17]. The findings suggest that adolescents with MDD may experience impairments in cardiopulmonary function and endurance performance compared to healthy adolescents, which could have important implications for treatment and intervention. To identify possible causes of impairment, the population of adolescents with MDD included in this analysis is examined in more detail. The high mean DIKJ score of the adolescents might indicate that they have low endurance performance. It could be shown that endurance training on the ergometer can significantly reduce the DIKJ score as well as that females have a higher DIKJ score [14]. Overall, 70% of the sample was female, reflecting the fact that young females are more likely to be affected by MDD than young males [43]. The mean body mass index (BMI) of females with MDD in this study is above the WHO normal mass range [44]. In addition, overweight and obese adolescents are more prone to MDD than normal mass individuals [45]. Furthermore, obesity reduces total lung capacity [46]. Since a large proportion of the female participants were overweight, BMI may be a relevant factor influencing CPET outcomes in the sample with MDD presented here. To better interpret the results, the relative CPET values are considered in the following.

The V̇O_2max_ is a widely accepted criterion to measure CRF. The lower values in adolescents with MDD may be explained by the fact that they usually have poor physical health and fitness levels, limited endurance training experience, restricted energy, and less motivation for maximal exercise effort [11]. One reason for this could be the low motivation to be active in extracurricular activities, which could be due to a poor social network and low support from the family [4].

The significantly lower mean V̇E_peak_ of adolescents with MDD is related to the low V̇O_2_ values [17]. Moreover, the value could be explained by the lack of cardiovascular regulation during a stressful situation, similar to the low HR [33, 36]. In addition, studies showed that adults as well as adolescents with MDD have lower overall physical fitness [33], which is equally highlighted in this analysis.

When V̇E increases excessively relative to the increase in V̇O_2_ and BLC rises slightly, VT1 is reached [46]. The VT1 is of great importance for predicting aerobic endurance performance as well as for prescribing training intensity in endurance sports [47]. Because only a few adolescents with MDD reached the BLC criterion, it can be assumed that the BLC did not increase significantly during exercise. Furthermore, participants had a lower mean V̇E_peak_ than the controls. Based on these rationales, the relatively low VT1 value can be inferred.

The gap between adolescents with MDD and sex- and age-related control values in performance was the largest when considering WR_peak_. The value can partly be explained by the different CPET protocols. The control values were collected while using a ramp protocol, whereas the participants in this study performed a step protocol. This was chosen since a loading protocol with a slowly increasing work rate is more effective for children and adolescents who are expected to have lower CRF due to a medical condition. Otherwise, premature test termination may occur [17]. Despite different loading protocols, only participants who achieved the exhaustion criteria were used for comparison with control values. Considering the mean WR_peak_ values of the two populations, the less performance of the adolescents with MDD can be explained by the lower physical activity [33]. This probably means a low skeletal muscle mass of the lower extremities, which has an influence on the WR_peak_ on the cycle ergometer [48].

Nevertheless, the different work rate increment protocols, as well as the inconsistent exhaustion criteria for a CPET in both groups may limit the comparability of this work. Another limitation is that there is not enough literature that applies the BLC criterion to adolescents, so in this case the ACSM recommended limit for adults was used [11]. Moreover, only four of the five ACSM exhaustion criteria were examined because subjectively perceived exertion was not considered. Besides, there is disagreement about the number of criteria that should be met, not only in adolescents with MDD but also in the healthy population [49].

In conclusion, this analysis indicates that a relevant proportion of adolescents with MDD do not achieve their V̇O_2max_ during a graded CPET. In addition to lack of motivation and reduced fitness levels, which may be associated with MDD, factors such as lower HR function, HR variability and overall BLC could also prevent achievement of the required exhaustion criteria. Besides, this work shows that adolescents with MDD have a significantly lower CRF compared to sex- and age-related control values.

In future studies, consistent and precise guidelines should be discussed regarding exhaustion criteria during a CPET. However, not only the number of criteria but also their content should be standardized for adolescents with MDD. Furthermore, it is important to adapt exercise protocols for CPET to the physical conditions of the participants to achieve the best possible and valid results.

The outcomes are relevant to clinical practice. Previous studies have already shown that physical activity reduces the symptoms of MDD [6, 8, 14]. Aerobic training has the greatest impact on improving depressive symptoms compared to strength or group training and can be used as an evidence-based treatment option [8, 9]. Therefore, this work should encourage CPETs to be performed as standard assessments in adolescents with MDD to determine the appropriate training intensity for therapeutic purposes. Next to the clinical relevance, the results should also prompt the social environment as well as policymakers to promote physical activity among adolescents to improve CRF and achieve a healthy body composition, which seems to be protective factors for the risk of MDD [12]. These include for example exercise opportunities in more places related to adolescent lives, such as schools or neighborhoods. In addition, this work should request an examination of exhaustion criteria during a CPET in adolescents with MDD in terms of number and content.

## Data Availability

The data that support the findings of this study are available on request from the corresponding author. The data are not publicly available due to privacy or ethical restrictions.

## Abbreviations

ACSM: American College of Sports Medicine
BLC: Blood lactate concentration
BLC_peak_: Peak blood lactate concentration
BMI: Body mass index
CPET: Cardiopulmonary exercise testing
RF: Cardiorespiratory fitness
DIKJ: Depressionsinventar für Kinder und Jugendliche
DSM-IV/-5: Diagnostic and Statistical Manual of Mental Disorders-Fourth-/-Fifth Edition
HR: Heart rate
HR_peak_: Peak heart rate
ICD-10: International Classification of Diseases-10th Revision
MDD: Major depressive disorder
RER: Respiratory exchange ratio
RER_peak_: Peak respiratory exchange ratio
SD: Standard deviation
TTE: Time to exhaustion
V̇CO_2_: Carbon dioxide production
V̇E: Minute ventilation
V̇E_peak_: Peak minute ventilation
V̇O_2_: Oxygen consumption
V̇O_2max_: Maximum oxygen consumption
V̇O_2peak_: Peak oxygen consumption
VT1: First ventilatory threshold
W: Watt
WHO: World Health Organization
WR: Work rate
WR_peak_: Peak work rate
